# Sampling Adipose and Muscle Tissue following Post-Harvest Scalding Does Not Affect RNA Integrity or Real-Time PCR Results in Market Weight Yorkshire Pigs

**DOI:** 10.3390/foods11121741

**Published:** 2022-06-14

**Authors:** Amy E. Bohan, Katelyn N. Purvis, Jason T. Sawyer, Werner G. Bergen, Terry D. Brandebourg

**Affiliations:** Department of Animal Sciences, Auburn University, Auburn, AL 36849, USA; aeb0005@auburn.edu (A.E.B.); katelyn.purvis@stjude.org (K.N.P.); jts0109@auburn.edu (J.T.S.); bergwg@auburn.edu (W.G.B.)

**Keywords:** pork quality, RNA integrity, real-time PCR, sampling time, scalding

## Abstract

Improving production efficiency while enhancing pork quality is pivotal for strengthening sustainable pork production. Being able to study both gene expression and indices of pork quality from the same anatomical location of an individual animal would better enable research conducted to study relationships between animal growth and carcass merit. To facilitate gene expression studies, adipose and muscle tissue samples are often collected immediately following exsanguination to maximize RNA integrity, which is a primary determinant of the sensitivity of RNA-based assays, such as real-time PCR. However, collecting soft tissue samples requires cutting through the hide or skin. This leaves the underlying tissue exposed during scalding, poses possible food safety issues, and potentially confounds pork quality measures. To overcome these limitations, the effect of tissue sample timing post-harvest on RNA integrity, real-time PCR results, and pork quality measurements was investigated by sampling subcutaneous adipose tissue and longissimus thoracis et lumborum muscle immediately following either exsanguination, scalding, or chilling. Sampling time did not affect RNA quality, as determined by the RNA integrity number of RNA samples purified from either adipose (RIN; *p* > 0.54) or muscle tissue (*p* > 0.43). Likewise, the sampling time did not influence the results of real-time PCR analysis of gene expression when comparing RNA samples prepared from adipose or muscle tissue immediately following either exsanguination or scalding (*p* > 0.92). However, sampling tissue prior to scalding resulted in a greater visual color score (*p* < 0.001) and lesser L* (*p* < 0.001) and b* (*p* < 0.001) values without impacting the 24 h pH (*p* < 0.41). These results suggested that if both RNA-based assays and meat quality endpoints are to be performed at the same anatomical location on an animal, tissue sampling to facilitate RNA-based assays should occur at a time point immediately following scalding. These findings demonstrated that sampling of adipose and muscle tissue can be delayed until after scalding/dehairing without decreasing the RNA integrity or altering the results of real-time PCR assays, while doing so was associated with little impact on measures of pork quality.

## 1. Introduction

Enhancing pork quality and production efficiency is pivotal for strengthening the sustainability of pork production [1,2,3]. Gene expression studies utilizing ribonucleic acid (RNA)-based assays, such as real-time PCR and RNAseq, are valuable tools for researchers to conduct mechanistic research of adipose and muscle biology and to better clarify how the factors that control the development of these tissues might also influence pork quality.

Correlating RNA-based and carcass merit data, however, presents a conundrum in research abattoirs. The sensitivity of RNA-based assays is determined by RNA integrity, which can be greatly influenced by factors related to tissue sampling [4,5,6,7,8,9,10]. Ribonucleic acids are highly susceptible to hydrolysis, both enzymatically by cellular and environmental ribonucleases (RNAses) and chemically in response to alkaline pH, rendering RNA very sensitive to degradation by postmortem processes and inadequate sample handling or storage [7,11,12,13]. Thus, to yield intact and pure RNA preparations that are representative of the physiological status of the tissue being studied, traditional sampling protocols dictate the rapid collection and preservation of tissue samples in ultra-cold conditions immediately after exsanguination [7,8,12].

On the other hand, cutting through the skin and leaving underlying tissue exposed before the carcass is scalded, de-haired, and further processed is likewise potentially problematic. For instance, such sampling presents potential food safety issues, promotes the separation of fat layers on the carcass during scalding, and potentially confounds the measurement of pork quality attributes, such as pH, color, and tenderness, near the sampling site due to the mechanical disruption of the tissue or through increased exposure to ambient temperature during subsequent carcass fabrication [14,15].

To address these issues, animal numbers are often increased to allow for some pigs to be used for tissue sampling, with the remaining pigs within a treatment being used for the measurement of carcass merit. Determining a sampling time that allows for optimized RNA integrity without confounding pork quality attributes would allow these endpoints to be measured within the same animal, greatly increasing the robustness of data while allowing for the use of fewer animals.

Importantly, the pH decline associated with the conversion of muscle to pork is in fact compatible with the goal of preventing alkaline conditions during RNA extraction [16,17,18]. Meanwhile, RNAse activity can be effectively controlled after sample harvest via the rapid processing and storage of tissue at ultra-cold temperatures, often in buffers that contain chaotic salts or a denaturing detergent [6,7,12]. This suggests that it may be possible to delay tissue sampling until a point in the pork harvest workflow whereby sampling would neither pose food safety issues, confound the estimation of pork quality parameters, nor impair the purification of high-quality RNA.

Therefore, the objective of this study was to determine whether tissue sampling could occur immediately following scalding/dehairing of the carcass without altering the results of subsequent RNA-based assays and pork quality measurements. We hypothesized that if sampling subcutaneous adipose and longissimus dorsi tissue after scalding was indeed appropriate, then no differences in RNA integrity, real-time PCR results, or pork color and ultimate pH would be attributable to the scalding process.

## 2. Materials and Methods

### 2.1. Animals and Design

**Figure 1:**
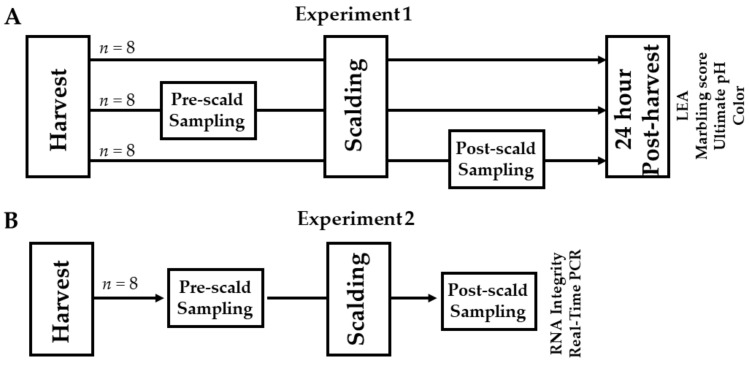
Schematic depicting the experiment design for experiment 1 (**A**) and experiment 2 (**B**).

All experimental procedures were reviewed and approved by the Auburn University Institutional Animal Care and Use Committee (IACUC; approval number: PRN 2012-2122). The Auburn University College of Agriculture is further accredited by the Association for Assessment and Accreditation of Laboratory Animal Care International (AALAC) and this study was conducted in accordance with the Federation of Animal Science Societies’ Guide for the Care and Use of Agricultural Animals in Research and Teaching. Purebred Yorkshire pigs (*Sus domesticus*; *n* = 32), sourced from the resident herd at the Auburn University Swine Research and Education Center (SREC), were reared according to typical industry practices to a body weight of 121.3 ± 3.6 kg. Figure 1 depicts the experimental design. Experiment 1 was conducted to determine the effects of timing of tissue sampling, i.e., pre-scalding versus post-scalding on indices of meat quality. To accomplish this aim, 24 pigs were reared to their target weights and then humanely harvested at the Auburn University Lambert Powell Meats Laboratory under inspection. Carcasses were randomly assigned to one of three groups: (1) a control group in which no tissue was sampled from the carcass, (2) a pre-scald group in which tissue was sampled from the carcass above the tenth rib on the right side immediately following exsanguination, and (3) a post-scald group in which tissue was sampled from the carcass above the tenth rib on the right side immediately following scalding/dehairing (5 min at 65 °C). Carcasses were then further processed as normal and pork quality measures were taken at the tenth rib on the right side of the chilled carcasses at 24 h post-harvest, as described below, to compare the impact that exposing the underlying tissue to scalding and air might have on the estimates of color.

Experiment 2 was conducted to determine the effects of timing of tissue sampling on markers of RNA integrity and the results of real-time PCR measurement of marker gene expression. To accomplish this aim, 8 additional pigs were reared and harvested as above. Tissue was sampled from above the 10th rib on the left side immediately following exsanguination (pre-scald) and then another tissue sample was collected from the same carcass above the tenth rib on the right side immediately following scalding/dehairing (post-scald) so that pairwise comparisons could be made on the same carcasses to determine the effect of timing of sampling on RNA integrity and PCR results.

In both experiments, samples of subcutaneous adipose tissue and longissimus muscle were collected by cutting a plug of tissue from the carcass of approximately 2.54 cm^2^ and extending from the skin through the loin muscle to approximately 3 cm deep above the tenth rib. Tissue samples were then immediately snap-frozen via immersion in liquid nitrogen within 5 min of collection and subsequently stored at −80 °C until RNA was later extracted and analyzed. Regardless of the timing of sample collection, carcasses were subjected to scalding and dehairing (immersion for 5 min at 65 °C) following exsanguination. All scalded carcasses were then processed according to standard operating procedures of the Lambert-Powell abattoir. Indices of meat quality were then measured at the tenth rib on the right side at 24 h postmortem as described below.

### 2.2. Carcass Analyses

The hot carcass weight was recorded after harvest and carcasses were chilled at 2 ± 1 °C for 24 h, at which point, carcasses were weighed again. At 24 h postmortem, carcass pH was recorded in the right loin using a portable pH spear probe meter (pH Spear, Oakton Instruments, Vernon Hills, IL, USA). Carcasses were then split between the 10th and the 11th ribs for evaluation of back fat (BF), loin eye area (LEA), subjective color of the loin, and degree of marbling. Longissimus muscles were also evaluated for objective color measurements at the 10th and 11th rib interface using a Hunter Miniscan XE Plus (Hunter Associates Laboratory, Reston, Virginia) for Hunter L*, a*, and b* values. The Miniscan was calibrated prior to use according to the manufacturer’s recommendations with a black and white tile, using a D 65 light source, a 10° viewing angle, and a 35 mm viewing area. Group means are reported as the average value of the indices sampled across 8 individual animals per group with each animal value representing the mean of three replicates within the animal.

### 2.3. Gene Expression Analysis

Total RNA for both pre- and post-scalding samples was extracted from adipose and muscle tissue using a two-step purification protocol with total RNA first being extracted from whole tissue using RNAzol^®^ RT (MRC, Inc., Cincinnati, OH, USA), followed by a second purification using RNAeasy spin columns (QIAGEN, Inc., Valencia, CA, USA) according to the manufacturers’ recommendations.

RNA was quantified and assessed for purity using a BioTek Synergy 4 plate reader utilizing the Take3 system (BioTek U.S., Winooski, VT, USA). Spectral scans ranging from 200 to 400 nm further verified the sample purity of all RNA samples as judged by the production of smooth curves exhibiting one peak at 260 nm. Total RNA integrity was accessed both (1) visually by resolving 2 μg of RNA on a denaturing formaldehyde gel stained with GelRed Nucleic Acid stain and (2) by determining an RNA Integrity Number (RIN) using an Agilent 2100 bioanalyzer (Agilent Technologies, Inc., Clara, CA, USA). All samples demonstrating sharp ribosomal bands with a 28S to 18S ratio estimated to be greater than one and RIN values greater than 7.0 were judged intact and non-degraded.

Total RNA was then reverse transcribed using 160 units of Superscript II reverse transcriptase (Promega Inc., Madison, WI, USA) and 0.5 μg Oligo(dT)_15_ primers in a reaction volume of 20 μL also containing 1 μg RNA/reaction, 6 mM MgCl_2_, 0.5 mM each of dNTP, and 20 units RNasin with the reaction being performed in a single cycle with the following steps: heating for 5 min at 65 °C, annealing for 5 min at 25 °C, elongation for 50 min at 42 °C, and heating for 15 min at 70 °C. The cDNA was subsequently stored at −80 °C until used in gene expression assays.

Real-time PCR was performed on the resultant cDNA using a Roche Lightcycler^®^ 480 Real-time PCR machine and LightCycler^®^ 480 SYBR Green I Master Mix (Roche Applied Science, Indianapolis, IN, USA) according to the manufacturer’s directions in a reaction volume of 20 μL consisting of 10 μL of master mix and 10 μL of H_2_O containing 50 ng of cDNA and 0.5 μM each of the forward and reverse primers under the following conditions: 1 pre-incubation step of 5 min at 95 °C followed by 45 cycles with each cycle consisting of a melting step of 10 s at 95 °C, and annealing step of 10 s at 54 °C, and an elongation step of 10 s at 72 °C. Each sample was run in three separate PCR runs with the resulting Cp values averaged across values obtained from three separate plates. All PCR reactions were performed using intron-spanning primers under optimized conditions with primer efficiencies ranging between 90 to 100% (Table 1), as verified with standard curves. All primers were designed to have an optimal annealing temperature of 54 °C. Product purity was assessed by melting curve analysis and expected amplicon sizes were verified on a 2% agarose gel stained with a GelRed Nucleic Acid stain. Values were normalized to the *S15* mRNA expression. The *S15* mRNA levels represented an appropriate control, as the efficiency of the *S15* primers was 100% (Table 1) and *S15* mRNA expression was not different between any groups tested (*p* > 0.05). Values were also normalized to *18S* and glyceraldehyde 3-phosphate dehydrogenase (*GAPDH*) mRNA expression as these also serve as common alternative control genes in the pig. Using neither *18S* nor *GAPDH* mRNA to normalize target gene expression altered the results compared to using *S15* as the control gene. Data are expressed as a fold change relative to baseline and calculated according to REST-MCS v2.0 [19,20,21]. Pre-scald and post-scald group means are reported as the average value of adipose or muscle tissue sampled across 8 individual animals per group, with each animal value representing the mean of three replicates from the animal.

### 2.4. Statistical Analysis

Changes in gene expression were calculated from the cycle threshold, after correction using S15 expression, and analyzed using the Pair Wise Fixed Reallocation Randomization Test of REST-MCS v2.0 (http://rest.gene-quantification.info/, accessed on 5 March 2022). Carcass traits and RNA quality data were analyzed as a completely randomized block design using a mixed linear model of SAS v9.2 (SAS Institute, Inc., Cary, NC, USA) with an individual animal serving as the experimental unit (i.e., individual block).

## 3. Results

### 3.1. Tissue Sampling Time Post-Harvest and Carcass and Pork Quality Traits

Effects of timing for tissue sampling on indices of pork quality were measured on chilled carcasses that were either not sampled (no sample control), were sampled prior to scalding (pre-scald sampling), or were sampled immediately following scalding (post-scald sampling). Pork quality traits were assessed 24 h post-harvest at the 10th rib on all chilled carcasses (Table 2). Neither the live weight (*p* > 0.93), hot carcass weight (*p* > 0.39), LEA (*p* > 0.33), nor marbling score (*p* > 0.71) differed between groups. The fresh pork ultimate pH was likewise uniform across groups (*p* > 0.57). However, both subjective and objective color scores differed with sampling time. Subjective color values were significantly darker in the pre-scald sampling group relative to carcasses that were either not sampled or those that were sampled following scalding (*p* < 0.0009). Likewise, objective instrumental color scores reflected a similar relationship. Both L* values (*p* < 0.0001) and b* values (*p* < 0.0001) were significantly lower in the pre-scald sampling group compared with carcasses within the control and post-scald sampling groups. Nonetheless, the sampling group did not alter the instrumental a* values (*p* > 0.81) of the collected pork tissue.

### 3.2. Tissue Sampling Time Post-Harvest and RNA Integrity and Real-Time PCR Estimates of mRNA Levels

To determine the effects of timing of tissue sampling on RNA integrity and the results of the RNA-based real-time PCR assay, an adipose and longissimus muscle tissue plug was removed from above the 10th rib on the left side of each carcass before scalding (pre-scald) and then again above the 10th rib on the right side of the same carcass immediately following scalding (post-scald) for all eight carcasses sampled. Total RNA was extracted from the tissue samples and analyzed for RNA integrity, both subjectively and objectively, before being used to measure mRNA abundance via real-time PCR. No differences in RNA integrity were detected subjectively when visualizing total RNA preparations via denaturing gel electrophoresis. All extracted total RNA samples were judged to be intact based upon (1) the presence of sharp ribosomal RNA (rRNA) bands with 28S rRNA that was approximately twice as prominent as 18S rRNA and (2) the general absence of smearing. No discernible differences between RNA samples were observed due to the time of sampling. Consistent with the subjective analysis, all electropherograms generated via capillary gel electrophoresis were indicative of fully intact RNA based upon the predominant abundance of large RNA fragments, as represented by the consistent presence of two predominant peaks associated with the two heavy ribosomal bands and the absence of short fragments below the 18S band. The RIN values ranged from 8.8 to 9.6 across all samples. Importantly, the electropherograms indicated that the time of sampling did not influence the integrity of the RNA preparations in any adipose or muscle samples analyzed. Likewise, RNA integrity did not differ by time of sampling in either the subcutaneous adipose (*p* > 0.54) or longissimus muscle tissue (*p* > 0.43) based upon the RIN values (Table 3).

**Figure 2:**
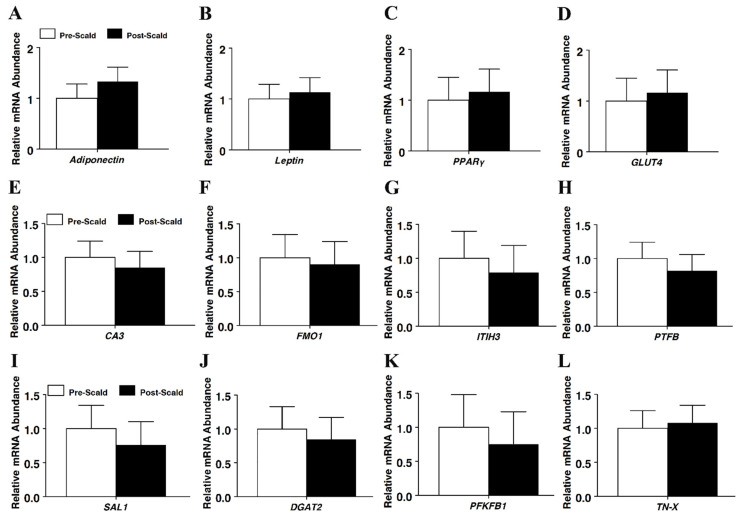
The effect of scalding on mRNA expression in subcutaneous adipose tissue of market weight hogs, as determined using real-time RT-PCR for genes (**A**) adiponectin, (**B**) leptin, (**C**) peroxisome proliferator-activated receptor gamma (*PPAR*γ), (**D**) glucose transporter 4 (*GLUT4*), (**E**) carbonic anhydrase III (*CA3*), (**F**) flavin-containing monooxygenase 1 (*FMO1*), (**G**) inter-alpha (globulin) inhibitor H3 (*ITIH3*), (**H**) 6-phosphofructo-2-kinase/fructose-2,6-biphosphatase 1 (*PFKFB1*), (**I**) salivary lipocalin (*SAL1*)*, (***J***)* diacylglycerol O-acyltransferase 2 (*DGAT2*), (**K**) pleiotropic factor beta (*PTFβ*), and (**L**) tenascin-X (*TN-X*). Values were normalized to *S15* mRNA abundance and expressed as a fold change relative to pre-scald samples and calculated according to Pfaffl (2010). Bars denoted by differ (*p* < 0.05); pre-scald, *n* = 8; post-scald, *n* = 8.

**Figure 3:**
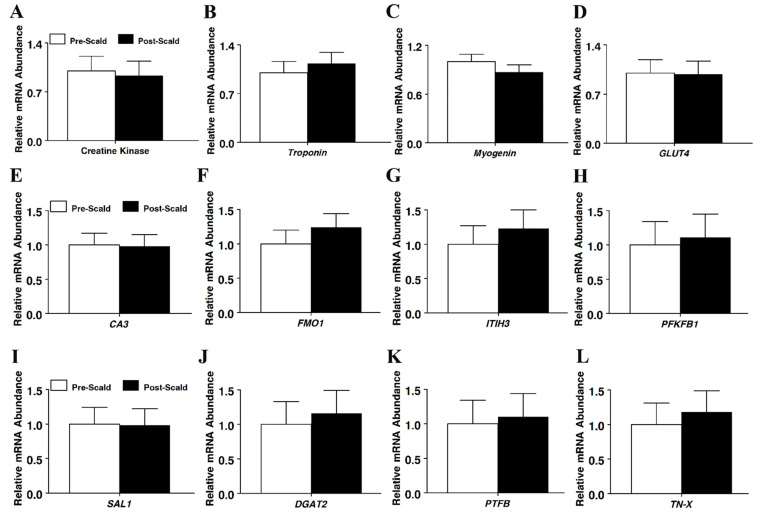
The effect of scalding on mRNA expression in the longissimus dorsi muscle of market weight hogs, as measured using real-time RT-PCR for genes (**A**) creatine kinase, (**B**) troponin, (**C**) myogenin, (**D**) glucose transporter 4 (*GLUT4*), (**E**) carbonic anhydrase III (*CA3*), (**F**) flavin-containing monooxygenase 1 (*FMO1*), (**G**) inter-alpha (globulin) inhibitor H3 (*ITIH3*), (**H**) 6-phosphofructo-2-kinase/fructose-2,6-biphosphatase 1 (*PFKFB1*), (**I**) salivary lipocalin (*SAL1*)*,* (**J**) diacylglycerol O-acyltransferase 2 (*DGAT2*), (**K**) pleiotropic factor beta (*PTFβ*), and (**L**) tenascin-X (*TN-X*). Values were normalized to *S15* expression. Data are normalized to S15 mRNA abundance and expressed as a fold change relative to pre-scald samples and calculated according to Pfaffl (2010). Bars denoted by differ (*p* < 0.05); pre-scald, *n* = 8; post-scald, *n* = 8.

RNA was further assessed using the RNA-based endpoint assay, namely, real-time PCR, to determine whether the timing of tissue sampling impacted estimates of mRNA abundance. The expression of the adipogenic marker genes adiponectin, leptin, peroxisome proliferator-activated receptor gamma (*PPAR*γ), and glucose transporter 4 (*GLUT4*) mRNA were not different between the pre- or post-scalded subcutaneous adipose samples (*p* > 0.05; Figure 2). Likewise, the expression of the myogenic gene markers creatine kinase, troponin, myogenin, and GLUT4 (Figure 3) also did not differ between sampling times (*p* > 0.05). Given that subcutaneous adipose tissue is more superficial anatomically than the underlying longissimus muscle tissue, the mRNA for several gene makers expressed by both adipose and muscle tissue were measured. The timing of tissue sampling did not alter the estimate of mRNA abundance of any such gene tested in either adipose tissue (Figure 2) or longissimus muscle (Figure 3), including the carbonic anhydrase III (*CA3*), flavin-containing monooxygenase 1 (*FMO1*)*,* inter-alpha (globulin) inhibitor H3 (*ITIH3*), 6-phosphofructo-2-kinase/fructose-2, 6-biphosphatase 1 (*PFKFB1*), salivary lipocalin (*SAL1*)*,* diacylglycerol O-acyltransferase 2 (*DGAT2*), pleiotropic factor beta (*PTFβ*), and tenascin-X (*TN-X*) genes.

## 4. Discussion

Appearance is an important selection criterion at the point of purchase and color is the most important appearance quality trait affecting the visible appeal of pork to consumers [22,23,24]. Thus, it is important both to study factors that may influence pork color and to avoid the potential that sampling carcasses during the harvest process to facilitate molecular studies into other quality traits, such as marbling, could confound or alter color measurements. To remove hair and ensure sanitary conditions during fabrication, the carcass was placed into a scalding vat containing an alkaline solution at a high temperature (≥60 °C). The scalding step during pork harvest could potentially confound color measurement in tissues exposed by cuts through the skin, while the high temperature and alkaline conditions could negatively impact the integrity of RNA within such exposed tissue. Given the necessity to better establish a post-harvest tissue sampling protocol where neither molecular nor quality endpoints are impacted by either the physical act of cutting into the carcass or the timing of collection, we sought to establish the effect of sampling prior to or following scalding on meat quality traits given the potential for scalding/dehairing to confound these measures.

Animals selected for this study were all of uniform, purebred Yorkshire genetics sourced from a high-health, biosecure herd. All were reared similarly from birth until harvest according to standard industry production practices, while no animal in this study was enrolled in prior research projects, eliminating the potential to confound these data herein. Importantly, the live weights and carcass traits, such as hot carcass weight, LEA, and marbling score, were uniform across all groups. This supported the hypothesis that any differences observed in indices of pork quality or RNA integrity in the present study were because of the timing of post-harvest sampling.

Sampling tissue before scalding resulted in significantly darker color scores. These data suggested that the act of cutting through the hide and removing a plug of adipose and muscle tissue from the carcass prior to scalding confounded the color measures by creating a perception of darker color in chops near the site of sampling. It is well established that temperature can affect the rate of pH decline during the conversion of muscle to meat and, as a result, impact color estimates of meat cuts [14,15,24]. Thus, cutting through the hide prior to scalding might be expected to alter 24 h post-harvest color estimates either through the exposure of the underlying tissue to the high temperature associated with scalding or due to increased exposure of the muscle to ambient air as muscle converts to meat during variable temperature environments associated with fabrication and chilling. Sampling the carcass following scalding prevented direct contact of the underlying tissue with the high temperature of the scalding vat; however, such a sampling strategy would not eliminate the possibility that exposure of the underlying tissue to the ambient air during chilling may influence the estimates of color. Given that the estimates of color were not different at the 10th rib between control carcasses and post-scald sampled carcasses in the present study, exposure of the underlying muscle to the high-temperature conditions of scalding appeared to be the critical confounding variable. Exposure of the underlying muscle to ambient air alone did not produce significant differences in color that others have observed in response to chilling [14,15,24]. Nonetheless, these data indicated that exposing the underlying muscle to the conditions common to scalding in abattoirs can result in a confounding perception of darker color 24 h post-harvest, which may not be reflective of the animal’s true carcass merit. These data support delaying tissue sampling until after scalding as best practice if pork quality and molecular data will be collected at the same anatomical site of a carcass.

Ribonucleic acids (RNAs) are highly susceptible to hydrolysis enzymatically by cellular and environmental ribonucleases (RNAses) and chemically in response to alkaline pH, rendering RNA very sensitive to degradation by postmortem processes and inadequate sample handling or storage [7,11,12,13]. Since the sensitivity and associated non-specific variation of RNA-based gene expression assays, such as real-time PCR, are heavily influenced by RNA quality, it is of paramount importance to optimize tissue sampling protocols to yield RNA preparations that are representative of the physiological status of the tissue being studied [12,16,17,18]. Given that RNAse activity is the most pernicious threat to RNA integrity, the rapid processing and storage of tissue at ultra-cold temperatures, often in buffers that contain chaotic salts, or a denaturing detergent represents the primary method of preventing RNA degradation [6,7,12]. However, the pH decline associated with the conversion of muscle to pork is compatible with the goal of preventing alkaline conditions during RNA extraction [16,17,18]. This suggests that it may be possible to delay tissue sampling until a point in the pork harvest workflow whereby sampling would neither confound the estimation of pork quality parameters nor impair the purification of intact RNA.

Automated capillary electrophoresis allows for the quantification of RNA integrity through the calculation of an RNA integrity number (RIN). RIN values approaching 0 represent completely degraded RNA and values approaching 10 represent RNA of the highest integrity [10]. This approach is currently considered a gold standard metric for accessing RNA integrity, with RIN values above 5 being considered suitable for real-time PCR applications, while values above 7 are generally considered a minimum requirement for omics approaches using microarray applications and next-generation sequencing technologies, such as RNAseq [8,18,25,26]. In the present study, all RIN values were near or above 9, regardless of the sampling time and no differences in RNA integrity were observed between the pre-scald and post-scald adipose or muscle tissue samples. These data indicated that RNA integrity was maintained post-harvest, at least through the scalding process, and that tissue sampling could be delayed until after scalding without detrimental effects on RIN.

Mammalian genes often display different sensitivities to RNA degradation (12). Furthermore, it is possible that the timing of sampling may impact RNA-based endpoint assays in a tissue-specific manner. Real-time PCR is a widely adopted method for quantifying mRNA levels from mammalian tissues [4,5,6,11,26,27]. In the present study, real-time PCR was used to measure the potential impact of scalding on estimates of mRNA abundance for a wide panel of both adipose and muscle-specific genes and multiple gene targets that are commonly expressed by both tissues. Given there was no reason to predict that gene expression levels would be significantly different between carcasses from animals that were genetically similar and uniformly reared, provided an appropriate sample size is adopted, we hypothesized that if scalding does confound RNA integrity or the results of RNA-based assays, then real-time PCR estimates of mRNA abundance would differ between the pre-scald and post-scald samples. Indeed, consistent with uniform RIN numbers across sampling times, no differences in apparent mRNA abundance were observed for any gene assayed in either the adipose or muscle tissue in the present study. This suggested that exposing the carcass to scalding did not confound the results of the real-time PCR assays. To our knowledge, this is the first study to examine the impact of scalding upon real-time PCR assays. These data provided no support for concerns that delaying sampling until after scalding might differentially impact mRNA abundances of genes in either adipose or muscle tissue. These results further supported the conclusion that delaying tissue sampling until after scalding represents best practice.

In the present study, total RNA was purified from sampled tissue using a well-established two-step extraction protocol that has been widely adopted due to the resulting yield of high-quality RNA preparations that can facilitate multiple RNA-based assays ranging from the use of real-time PCR to measure mRNA expression of a single target gene to RNAseq analysis of entire transcriptomes [8,28]. However, small oligonucleotides of 200 base pairs or less are inefficiently captured when passing the RNA preparations over a spin column and this could result in an underestimate of RNA sample degradation [28,29]. In the current study, no signs of RNA degradation were observable when visualizing RNA samples via denaturing gel electrophoresis. Importantly, there were no differences in RNA integrity or estimates of mRNA abundance across sampling groups based upon RIN values and the results of real-time PCR analysis of a wide panel of gene targets in both adipose and muscle tissue. While the occurrence of some degree of scalding-associated RNA degradation cannot be definitively ruled out in the present study, there was no evidence that such degradation did occur or was substantial enough to confound the results of RNA-based assays, such as real-time PCR.

## 5. Conclusions

In conclusion, the effect of timing for tissue sampling on RNA quality, adipose and muscle tissue marker gene expression, and pork quality indices, such as color and ultimate pH, was investigated by comparing the effects of harvesting tissue at the tenth rib either immediately following exsanguination or immediately following scalding of the carcass. The sampling time did not affect the RNA quality or alter the expression of adipose or muscle genes. However, sampling tissue prior to scalding resulted in the perception of darker color in the muscle near the sampling site. These data indicated that delaying the timing of sampling until after scalding/dehairing posed no threat to optimal RNA integrity and subsequent analysis with RNA-based assays. Specifically, sampling subcutaneous adipose and muscle tissue immediately following scalding/dehairing during carcass fabrication facilitated the purification of high integrity total RNA preparations that were suitable to allow RNA-based assays to be conducted without impacting the meat quality endpoints. Furthermore, these data supported a best practice tissue sampling protocol, which allowed molecular endpoints and pork quality indices to be measured at the same anatomical location of individual carcasses. One limitation of the present study centered upon the use of total RNA and real-time PCR to assess RNA integrity. Recently non-coding RNA, such as microRNA and long non-coding RNA species, were implicated in regulating important developmental events in mammals. Future studies will aim to extend our present findings to characterize the impact of the timing of tissue sampling on non-coding RNA species. Nonetheless, this improved sampling strategy allowed for the use of fewer animals while increasing the fidelity of data in studies aimed at better understanding links between the regulation of adipose and muscle development and carcass merit in pigs.

## Figures and Tables

**Table 1:** Oligonucleotide polymerase chain reaction primers.

Gene	Accession No.	Primer sequence (5′→3′)	Orientation	Efficiency
***S15***	NM_214334.1	GGTAGGTGTCTACAATGGCAAGG	Forward	100%
		GGCCGGCCATGCTTC	Reverse	
***PPARγ***	NM_214379.1	AATTAGATGACAGCGACCTGGCGA	Forward	96%
		TGTCTTGAATGTCCTCGATGGGCT	Reverse	
***GLUT4***	NM_001101031.2	TCCTCTGAGCCTTTCCAGCAAGTT	Forward	93%
		ACCATCAGCATTCGGTTACCAGGA	Reverse	
***Adiponectin***	NM_214370.1	TCTCGGCCAGGAAACCACCGA	Forward	100%
		CGGCCTGGGGTACCGTTGTG	Reverse	
***Leptin***	NM_213840.1	ACGATTGTCACCAGGATCAG	Forward	92%
		ACAAACTCAGGACAGGATGG	Reverse	
***Creatine kinase***	NM_001129949.1	GCTCCAAGACACTCATCCAC	Forward	95%
		TTGCGTAGATCAGGGTAGTC	Reverse	
***Troponin***	NM_001001862.1	GAGATGATCGCTGAGTTCAAGG	Forward	90%
		GATGTAGCCGTCCATGTTCC	Reverse	
***Myogenin***	NM_001012406.1	GTAAGAGGAAGTCCGTGTCTG	Forward	91%
		TGTGGGAACTGCATTCACTG	Reverse	
***CA3***	NM_001008688.1	AAATTAAGACAAAGGGCAAGGAGG	Forward	91%
		CAGGATGGGTTGAAGTTCGT	Reverse	
***FM01***	NM_214064.1	CGAAGACAGGATACAACTAAGAG	Forward	100%
		ACTTTCCCAGTGATGATACGG	Reverse	
***ITIH3***	NM_001206349.1	GAAGAAACAGCCCAACCACC	Forward	93%
		GAAGAATTGTCCCAGAAGCCC	Reverse	
***PFKFβ1***	NM_001143721.1	ACTACGAATTCTTTCTCCCAGAC	Forward	97%
		CACACTGCTTCCTTATAAGTAGG	Reverse	
***SAL1***	NM_213814.1	AAGCAGGCCAAGATGTTGTG	Forward	91%
		CACTCTCCGTTTACCTTTCTCTG	Reverse	
***DGAT2***	NM_001160080.1	CGAGACTACTTTCCCATCCA	Forward	99%
		GAACTTCTTGCTCACTTCGG	Reverse	
***PTFβ***	NM_214336.1	GAAGACAGTCACCATCTCCA	Forward	100%
		TAGATTCTGCTTGAGGTTTGGG	Reverse	
***TN-X***	NM_001123204.1	ACACCTTTGACCACTTCCAG	Forward	100%
		CATCCCTGTCCATAATGCCT	Reverse	

**Table 2:** Carcass and pork quality traits by the timing of the tissue sampling group ^1,2^.

Variable	No Sample Control	Pre-Scald Sampling	Post-Scald Sampling	*p*-Value
Live wt, kg	123.7 ± 3.6	119.5 ± 3.3	120.3 ± 2.6	0.93
Hot carcass wt, kg	78.5 ± 2.6	75.6 ± 2.7	76.6 ± 2.4	0.39
LEA ^3^, cm ^2^	6.94 ± 0.27	7.19 ± 0.28	7.23 ± 0.23	0.33
Marbling score ^4^	1.20 ± 0.10	1.19 ± 0.14	1.09 ± 0.04	0.71
Ultimate pH ^5^	5.84 ± 0.02	5.89 ± 0.05	5.83 ± 0.07	0.57
Color ^6^	2.40 ± 0.13 ^a^	3.09 ± 0.16 ^b^	2.31 ± 0.12 ^a^	0.0009
L*, lightness	64.8 ± 0.3 ^b^	55.4 ± 0.8 ^a^	65.9 ± 0.9 ^b^	0.0001
a*, redness	10.3 ± 0.2	10.3 ± 0.2	10.4 ± 0.2	0.81
b*, yellowness	18.1 ± 0.2 ^b^	16.1 ± 0.2 ^a^	18.5 ± 0.3 ^b^	0.0001

^1^ Values are means ± SEM; group means within a row with different superscripts differ (*p* < 0.05), *n* = 8; ^2^ *No sample control*: carcasses that did not have tissue removed prior to scalding, *pre-scald sampling*: a 2.54 cm^2^ plug was sampled on the right side at the tenth rib prior to scalding and dehairing, *post-scald sampling*: a 2.54 cm^2^ plug was sampled on the right side at the tenth rib immediately following scalding/dehairing; ^3^ loin eye area: measured 24 h post-harvest on chilled carcasses; ^4^ subjective marbling score: 1 to 2.4—devoid, 2.5 to 4—traces, 4 to 5—slight; ^5^ ultimate pH: measured 24 h post-harvest on chilled carcasses; ^6^ visual (subjective) color score: five-point scale where 1—very light and pale, 5—dark red, etc.

**Table 3:** RNA integrity number by sampling group in total RNA samples extracted from subcutaneous adipose and longissimus dorsi muscle tissue ^1,2^.

Tissue	Pre-Scald	Post-Scald	*p*-Value
Subcutaneous adipose	9.1 ± 0.2	9.4 ± 0.2	0.54
Longissimus dorsi muscle	9.0 ± 0.2	9.1 ± 0.2	0.43

^1^ Values are means ± SEM; ^2^ pre-scald and post-scald samples were harvested from the same carcass for a pairwise comparison of the effect of timing of sampling within the same animal with pre-scald tissue taken above the 10th rib on the left side immediately following exsanguination and post-scald tissue taken above the 10th rib on the right side immediately following scalding.

## Data Availability

The data presented in this study are available on request from the corresponding author. The data are not publicly available due to Auburn University data security and management policy.
